# Inhibition of G9a induces DUSP4-dependent autophagic cell death in head and neck squamous cell carcinoma

**DOI:** 10.1186/1476-4598-13-172

**Published:** 2014-07-15

**Authors:** Kai-Chun Li, Kuo-Tai Hua, Yi-Shen Lin, Chia-Yi Su, Jenq-Yuh Ko, Michael Hsiao, Min-Liang Kuo, Ching-Ting Tan

**Affiliations:** 1 Graduate Institute of Toxicology, College of Medicine, National Taiwan University, Taipei, Taiwan; 2 Genomics Research Center, Academia Sinica, Taipei, Taiwan; 3 Department of Otolaryngology, National Taiwan University Hospital and National Taiwan University College of Medicine, Taipei, Taiwan; 4 Graduate Institute of Biomedical Sciences, College of Life Science, National Taiwan University, Taipei, Taiwan

**Keywords:** HNSCC, G9a, autophagy, DUSP4, ERK

## Abstract

**Background:**

Head and neck squamous cell carcinoma (HNSCC) is a common cancer worldwide. Emerging evidence indicates that alteration of epigenetics might be a key event in HNSCC progression. Abnormal expression of histone methyltransferase G9a, which contributes to transcriptional repression of tumor suppressors, has been implicated in promoting cancerous malignancies. However, its role in HNSCC has not been previously characterized. In this study, we elucidate the function of G9a and its downstream mechanism in HNSCC.

**Methods:**

We investigated the clinical relevance of G9a in HNSCC using immunohistochemistry (IHC) staining. In vitro cell proliferation and tumorigenesis ability of G9a-manipulated HNSCC cells were examined with MTT assays, clonogenic assays, and soft agar assays. We examined different routes of cell death in HNSCC cells induced by G9a-depletion or enzymatic inhibition by immunoblot, flow cytometry, fluorescent and transmission electron microscopy analysis. Specific targets of G9a were identified by affymetrix microarray and quantitative reverse transcription-polymerase chain reaction (qRT-PCR). Lastly, functions of G9a in vivo were confirmed with a xenograft tumor model.

**Results:**

G9a expression is positively correlated to proliferation marker Ki-67 and to poor prognosis in HNSCC patients. Genetic or pharmacological inhibition of G9a reduced cell proliferation without inducing necrosis or apoptosis. Instead, autophagic cell death was the major consequence, and our investigation of mechanisms suggested it is mediated via the dual specificity phosphatase-4 (DUSP4) dependent ERK inactivation pathway. An orthotopic tumor model further confirmed the growth inhibiting effect and induction of autophagy that followed suppression of G9a.

**Conclusions:**

In this study, we provide evidence that G9a confers the survival advantage of HNSCC. Genetic or pharmacological inhibition of G9a induces autophagic cell death; this finding provides a basis for new therapeutic targets for treating HNSCC.

## Background

Head and neck squamous cell carcinoma (HNSCC), including squamous cancers of the oral cavity, pharynx and larynx, is one of the common cancers, estimated to account for over 300,000 deaths yearly worldwide; in Taiwan, it is the fifth most common malignancy. Although improved treatment strategies for HNSCC are continually being proposed, clinical outcomes have not improved noticeably [1,2]. Therefore, identifying and developing effective new therapeutic strategies remains a matter of great urgency for clinical management of HNSCC.

DNA methylation is an epigenetic effect of gene regulation. Recent studies have proposed that DNA methylation on the genes promoter region is controlled by post-translational modification of histone proteins [3]. Among different states of histone modification, methylation is generally considered an important mechanism for regulating gene expression and chromosome organization that may contribute to development and maintenance of physiological functions [4,5]. In mammals, methylation on histone 3 lysine 9 (H3K9) is essential for transcriptional repression. Mono- (H3K9me1) and di-methylation of H3K9 (H3K9me2) are mainly mediated by G9a (a histone methyltransferase), which is located predominantly at the euchromatin [6]. Accumulating evidence demonstrates that G9a is up-regulated in various types of cancer. Dysregulation of G9a triggers an abnormal H3K9me2 expression pattern, resulting in silencing of tumor suppressor genes [7-11].

In carcinogenesis, G9a has been reported to promote both cell proliferation and metastasis [7,10]. Knockdown of G9a by RNAi mediated gene silencing inhibits growth and further promotes apoptosis of breast cancer cells [8,12]. Pharmacological inhibition of G9a disturbs the cell cycle and induces senescent phenotypes, further inhibiting growth in prostate and pancreatic cancer [13,14]. Furthermore, inhibition of G9a may lead to autophagy in breast and colorectal cancers [15], suggesting that G9a may control cell growth in a variety of cancer types via multiple routes. However, its role in HNSCC and the underlying mechanisms remain largely unknown.

Autophagy is a physiological process that turns over constituents of eukaryotic cells. It is initiated by vesicle fusions and forms the double-membraned autophagosomes. The autophagosome will then fuse with lysosome to degrade the proteins or organelles [16]. Although autophagy was initially considered a temporary survival mechanism for normal and cancer cells under conditions of nutrient limitation and metabolic stress, several studies have shown that persistent stress may lead to an intensification of autophagy that results in cell death, which is called type II programmed cell death [17]. These findings provide a basis for identifying the key regulator mediating autophagy-associated cell death and elucidating its mechanism, may be benefit for developing cancer therapies.

This study reveals the importance of G9a in HNSCC. Its expression is up-regulated in tumors and corresponds strongly with poor prognosis in clinical practice. We found that knockdown of G9a or pharmacological inhibition with a specific chemical inhibitor significantly reduced proliferation, colony formation and anchorage-independent growth, and the mechanism is not mediated by induction of necrosis or apoptosis, but regulated by enhancement of autophagy. Moreover, G9a-induced autophagic cell death in HNSCC was mainly mediated by dual specificity phosphatase-4 (DUSP4) dependent ERK inactivation. Our findings shed light on the role of G9a in regulation of cell growth in HNSCC, which may provide a valuable therapeutic strategy for treating such cancers.

## Results

### G9a expression is associated with proliferation and poor prognosis in HNSCC

**Figure 1:**
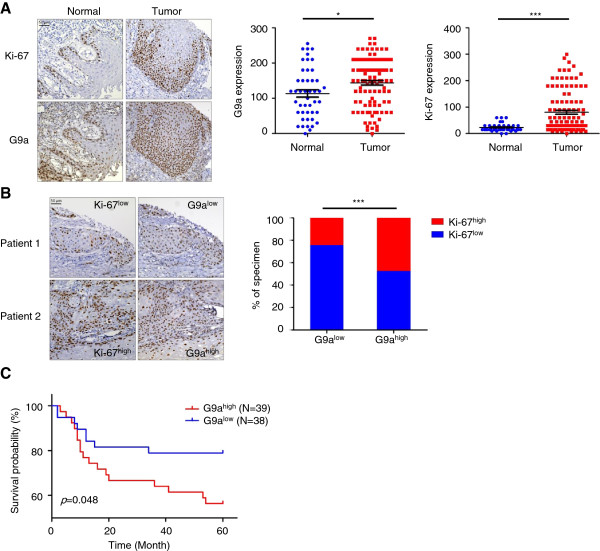
**G9a expression is positively correlated with cell proliferation and poor prognosis in HNSCC patients. (A)** Correlation between Ki-67 and G9a expression. The expression of two proteins were analyzed by IHC staining of tissue microarray (TMA) containing 46 normal and 108 tumor sections from HNSCC patients. The expression level of both Ki-67 and G9a was measured by relative staining intensity multiplied by percentage. Scale bar, 50 μm. (*, *p* < 0.05; ***, *p* < 0.001). **(B)** Ki-67 and G9a IHC staining of serial section of tumors. Low or high Ki-67 and G9a expression were defined by a threshold of 80 and 143, which is the average of relative staining intensity multiplied by percentage of Ki-67 and G9a. Scale bar, 50 μm. (***, *p* < 0.001). **(C)** Kaplan-Meier plot of overall survival of 77 HNSCC patients from a National Taiwan University Hospital cohort stratified by G9a expression level.

It has been suggested that G9a controls cells growth in various types of cancer. To investigate whether G9a expression is also involved in HNSCC growth and its clinical implications, we first analyzed the expression of G9a and proliferation marker, Ki-67, by IHC staining of a tissue microarray (TMA) containing a cohort of 108 HNSCC specimens. In the normal mucosa, G9a and Ki-67 staining were both localized predominantly in highly proliferative basal cells (Figure 1A). Both G9a and Ki-67 were expressed at a higher level in tumor tissues compared with adjacent normal mucosa (Figure 1A). Moreover, a similar expression pattern was observed for G9a and Ki-67 (Figure 1B, Additional file 1: Table S2), suggesting that G9a may also be involved in regulating growth in HNSCC. To further elucidate the clinical significance of G9a, we have collected 77 specimens from an independent HNSCC cohort and examined the correlation between G9a expression and survival probability. Patients with high G9a scoring tumors (scores 2 and 3) had a significantly worse prognosis compared with those whose G9a staining score was lower (scores 0 and 1) (Figure 1C). Taken together, this indicates that G9a may regulate cell proliferation in HNSCC and could also be an indicator for predicting clinical outcomes of HNSCC patients.

### Inhibition of G9a attenuates HNSCC cell growth in vitro

**Figure 2:**
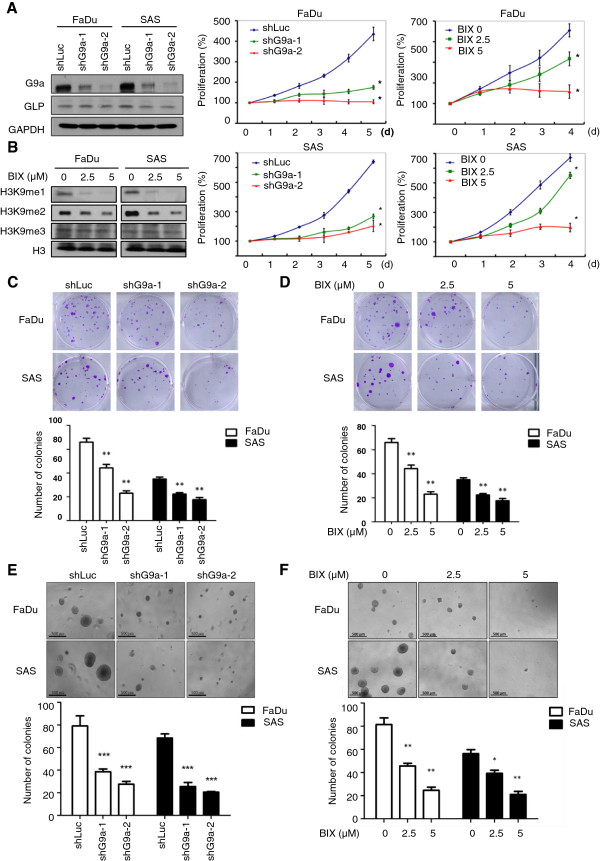
**Knockdown of G9a or inhibition of its enzymatic activity decreases HNSCC cell growth in vitro. (A)** FaDu and SAS cells were infected with lentivirus containing two different G9a-shRNA plasmids to knockdown G9a expression. **(B)** Cells were treated with different doses of BIX-01294 to inhibit the enzymatic activity of G9a. We used MTT assay to determine the five-day proliferation rate of G9a-knockdown cells compared with luciferase-knockdown controls, and BIX-01294 treated cells compared with vehicle (*, *p* < 0.05). The MTT value at Day 0 was considered as 100% of the proliferation rate for each experimental group. (**C** and **D**) The clonogenic cell survival assay of cells infected with lentivirus containing luciferase shRNA, G9a shRNA, vehicle and BIX-10294 treated cells (**, *p* < 0.01). (**E** and **F**) Examination of anchorage-independent growth ability of cells and quantification results of soft agar assay. Scale bar, 500 μm. (*, *p* < 0.05; **, p < 0.01; ***, *p* < 0.001).

To elucidate the relationships between G9a expression and proliferation in HNSCC, we first examined the growth of two human HNSCC cell lines, SAS and FaDu, with G9a silencing by two independent shRNAs. The G9s shRNAs specifically deplete G9a but not GLP protein expression (Figure 2A), which is a G9a-like protein and can form a heteromeric complex with G9a to catalyze H3K9 methylation in cells [18]. As shown in Figure 2A, the proliferation rates were significantly decreased within five days after inhibition of G9a expression. A similar effect was also observed with pharmacological inhibition of G9a in HNSCC cells by BIX-01294 treatment in a dose-dependent manner (Figure 2B). The BIX-01294 treatment decreased H3K9 mono- and di-methylation rather than tri-methylation, suggesting a highly G9a-specific inhibition of this compound. Given the essential role of G9a in regulating HNSCC growth in vitro, we then determined whether G9a could regulate colony formation, in order to evaluate the capacity of a single cell to expand. The results clearly indicated that cells with G9a knockdown (Figure 2C) or inhibitor treatment (Figure 2D, Additional file 2: Figure S1A and B) produced smaller colonies than vehicle control. The results for anchorage-independent growth ability measured by soft agar assay also showed that inhibition of G9a decreased the size and number of colonies (Figure 2E and F). Moreover, the BrdU incorporation assay was performed to detect new synthesized DNA during cell proliferation. As shown in Additional file 3: Figure S2, cells with genetic inhibition (Additional file 3: Figure S2A) or pharmacological inhibition (Additional file 3: Figure S2B) of G9a displayed less BrdU incorporation compared to the vehicle counterpart, suggesting that G9a may positively regulate cell growth in HNSCC.

### Inhibition of G9a attenuates HNSCC growth, but not via induction of apoptosis or necrosis

**Figure 3:**
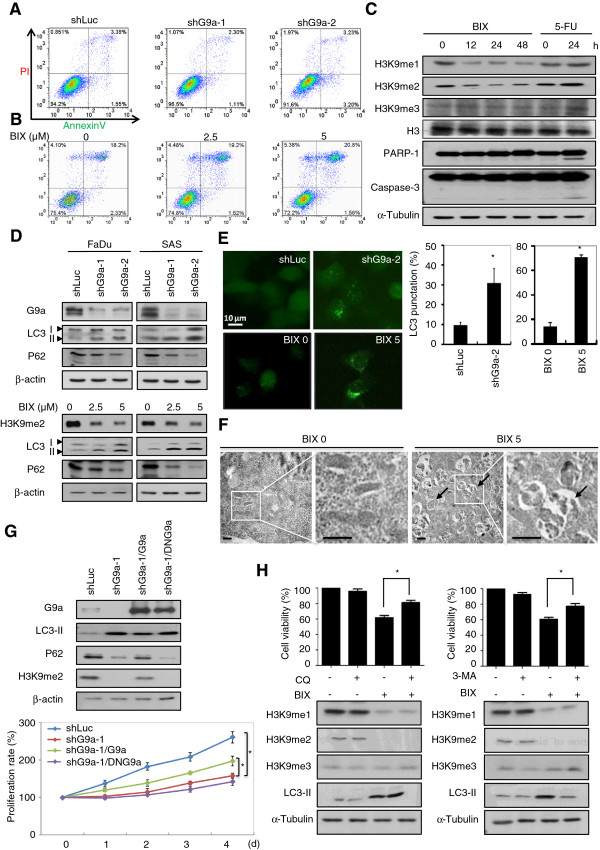
**Inhibition of G9a induces autophagy-dependent death in HNSCC cells. (A)** Flow cytometry analysis of apoptosis and necrosis of FaDu cells with control or G9a-knockdown group. **(B)** Flow cytometry analysis of apoptosis and necrosis of vehicle or FaDu cells treated with 5 μM BIX-01294. **(C)** Western blot analysis of H3K9 methylations and apoptosis markers (PARP and caspase-3) expression in drug-treated FaDu cells. α-Tubulin is shown as an internal control. **(D)** Western blot analysis of G9a, H3K9me2, autophagy marker p62 and LC3 expression in G9a-knockdown or BIX-01294 treated cells. β-actin is shown as an internal control. **(E)** Photograph of LC3-GFP expression in FaDu cells and the punctuation quantification. Scale bar, 10 μm. Values are presented as mean ± SD of 5 different fields in each group. A significant difference was observed between the groups (**, *p* < 0.01). **(F)** Examination of autophagosome formation by transmission electron microscopy. Arrows denote the autophagosome structure in the cytoplasm of FaDu cells. Scale bar, 1 μm. **(G)** Western blot analysis of autophagic marker expression in FaDu cells with G9a-knockdown and restored with wild-type G9a or dominant-negative G9a (DN-G9a). The proliferation rate was measured by MTT assay (*, *p* < 0.05). **(H)** FaDu cells were treated with DMSO or 5 μM BIX-01294 in the presence or absence of 2 mM 3-MA or 100 μM chloroquine. Cell viability was measured by MTT assay 24 hours later.

To further explore the mechanism of inhibiting cell growth after knockdown of G9a or blocking its enzymatic activity, we then investigated whether inhibition of G9a could induce apoptosis or necrosis in HNSCC cells. The Annexin V and PI binding assay was performed and analyzed by flow cytometry. As shown in Figure 3A, no significant apoptotic or necrotic cells were detected 72 hours after knockdown of G9a compared with the luc shRNA control. In addition, there was no induction of apoptosis and necrosis in cells subjected to 5 μM BIX-01294 treatment for 24 and 48 hours (Figure 3B). We then performed biochemical analysis by immunoblotting with pro-apoptotic markers, including cleavage forms of the poly ADP-ribose polymerase (PARP) and caspase-3. Consistently, we were unable to detect pro-apoptotic signatures in cells within 48 hours of treatment with BIX-01294 (Figure 3C). A previous study demonstrated that 5-Fu treatment induced sub-G1 populations of HNSCC [19]. Here, the cytotoxic effect of 5-Fu was shown by induction of apoptosis in HNSCC cells, demonstrating that the apoptosis mechanism is intact in HNSCC cells, a finding which further suggested that inhibition of G9a decreases cell growth via a non-apoptotic process.

### Autophagic cell death is the major consequence of G9a inhibition in HNSCC cells

While investigating the growth inhibition effect, we observed obvious vacuoles in the cytoplasm within G9a knockdown or BIX-01294 treated HNSCC cells. This morphologic change resembles autophagy. Autophagy is a cellular-catabolic mechanism in eukaryotic cells, which sequesters cytoplasmic proteins or organelles into membrane-bound autophagosomes and then the autophagosomes would be transported to lysosome for degradation, both during homeostasis and cellular dysfunction [16,17,20]. We therefore investigated whether inhibition of G9a induced autophagy in HNSCC cells. Biochemical results suggested that knockdown or pharmacological inhibition of G9a increased cleaved LC3 (LC3-II) expression, which is an autophagy marker essential for triggering autophagosome formation [21]. Furthermore, the expression level of p62/SQSTM1, an adapter protein of LC3 binding to ubiquitin [22], was reduced (Figure 3D and Additional file 2: Figure S1A). To gain further insight into the autophagy mechanism triggered by inhibition of G9a, we then used a fluorescence microscope to examine autophagosome formation using FaDu cells stably transfected with GFP-LC3 encoded plasmid. As shown in Figure 3E, knockdown of G9a for 72 hours or treatment with its inhibitors for 24 hours significantly increased LC3 punctuation compared with control cells. Furthermore, electron microscopy clearly displayed the presence of autophagosomes in the cytoplasm (Figure 3F).

To directly examine the role in HNSCC cells of G9a-mediated H3K9 methylations in autophagy, we then restored HA-tagged wild type or catalytically defective form of G9a (DN-G9a), containing N903H and L904E mutations in the SET domain [23], into FaDu cells with G9a knockdown. As shown in Figure 3G, rescuing the cells with wild-type G9a reduced LC3-II but increased p62 expression. Moreover, the cell proliferation rate was also partially restored after G9a re-expression. However, DN-G9a showed no reverse effects on autophagy markers expression and cell proliferation rate (Figure 3G), which suggests that the enzymatic activity of G9a exerts a key role in mediating autophagy in HNSCC.

Dual functions of autophagy have been previously proposed: autophagy-dependent cell survival and autophagy-associated cell death [16]. Pre-treatment to restrain autophagy with lysosome acidification inhibitor chloroquine (CQ) attenuated the inhibition effect on cell viability induced by BIX-01294 (Figure 3H, left). A similar effect was also observed in cells with 3-MA (3-methyladenine) addition, an autophagic sequestration blocker (Figure 3H, right). Together, these findings indicate that the genetic or pharmacological inhibition of G9a that causes growth arrest is worked mainly via activation of the autophagic cell death mechanism.

### Inhibition of G9a activates DUSP4-dependent ERK dephosphorylation to further induce cellular autophagy

**Figure 4:**
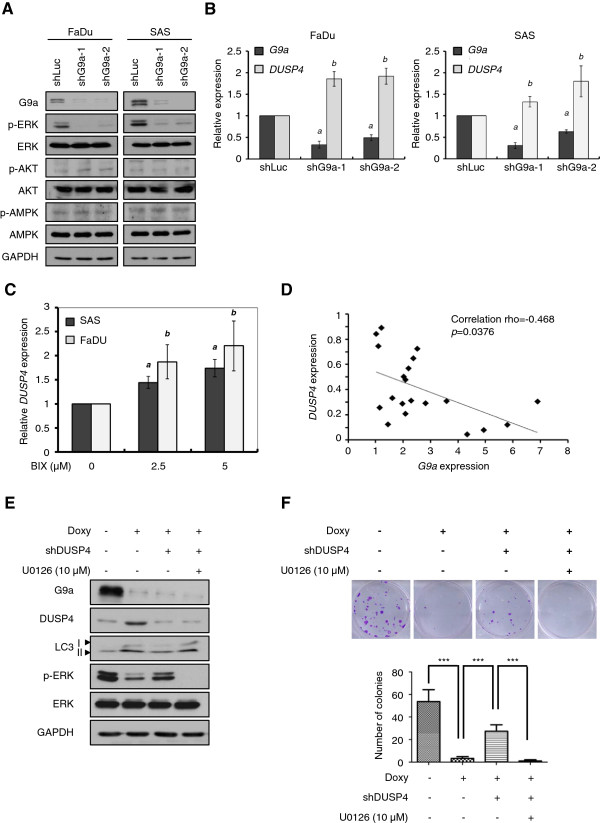
**Inhibition of G9a induces the DUSP4-ERK-mediated autophagy mechanism in HNSCC cells. (A)** Lysates from control or G9a-knockdown cells of FaDu or SAS cells were subjected to western blot analysis for examination of autophagy-related signaling pathways. **(B)** Quantitative real-time PCR analysis of *G9a* and *DUSP4* expression in control or G9a-knockdown FaDu or SAS cells. *a*, *G9a* expression in G9a-knockdown cells compared with shLuc-containing lentivirus-infected controls. *b*, *DUSP4* expression in G9a-knockdown cells compared with shLuc-containing lentivirus-infected controls, *p* < 0.01. **(C)** Quantitative real-time PCR analysis of *DUSP4* expression in controls or BIX-01294 treated FaDu or SAS cells for 24 h. *a*, *DUSP4* expression in SAS cells treated with 2.5 or 5 μM BIX-01294 compared with untreated controls. *b*, *DUSP4* expression in FaDu cells treated with 2.5 or 5 μM BIX-01294 compared with untreated controls, *p* < 0.05. **(D)** The spearman correlation between G9a and DUSP4 mRNA expression of tumors from 20 HNSCC patients. **(E)** Cell lysates from FaDu cells expressing a combination of inducible-knockdown of G9a, transient knockdown of DUSP4 and 10 μM U0126 treatment were subjected to western blot to analyze the indicated proteins. **(F)** The clonogenic cell survival assay of FaDu cells expressing a combination of inducible-knockdown of G9a, transient knockdown of DUSP4 and 10 μM U0126 treatment. Significant differences were determined between the groups (***, *p* < 0.001).

To understand the underlying mechanism in the autophagy process mediated by G9a inhibition, we examined several signal transduction pathways involved in autophagy [24-26]. In both FaDu and SAS cells, ERK phosphorylation was dramatically decreased in G9a-knockdown cells (Figure 4A). In order to clarify whether inactivation of ERK is the major autophagy mechanism induced by G9a inhibition, we examined S-6-kinase (p-S6K) protein phosphorylation on T389 residue, which has been demonstrated as dependently activated by ERK-mTOR signaling [27]. As shown in Additional file 4: Figure S3, p-S6K was reduced in FaDu cells treated with BIX-01294, which suggests that ERK inactivation may play an important role in autophagy caused by G9a inhibition. Given the role of G9a in transcriptional control of gene expression, we then performed affymetrix microarray analysis to identify the downstream target correlated with autophagy induction. We identified a total of 2,326 genes ≥ 1.5-fold upregulated in FaDu cells with G9a-knockdown. Furthermore, an ERK de-phosphatase, dual specificity phosphatase-4 (*DUSP4*) showed more than 2-fold up-regulation (GSE56330). We confirmed the expression of *DUSP4* by real-time PCR analysis in two HNSCC cell lines with genetic or pharmacological inhibition of G9a (Figure 4B and C). Moreover, we observed a negative correlation with significance between *G9a* and *DUSP4* in 20 HNSCC tumor specimens (Figure 4D). To address whether G9a-inhibition induced DUSP4 activation may contribute to ERK inactivation and autophagy, we introduced a doxycycline (Dox)-inducible shG9a transgene (pTRIPZ-shG9a) into FaDu cells and established a stable clone by puromycin selection. The results revealed that Dox treatment combined with DUSP4 knockdown suppressed LC3-II expression and reversed colony formation, compared with cells receiving Dox treatment alone. Furthermore, ERK activation was then abolished. Remarkably, addition of ERK phosphorylation inhibitor U0126 compensated for the effects in cells with DUSP4 and G9a knockdown (Figure 4E and F), demonstrating that autophagy induced by G9a inhibition was mainly mediated through the DUSP4-dependent ERK inactivation mechanism.

### Inhibition of G9a causes autophagy and suppresses tumor growth in vivo

**Figure 5:**
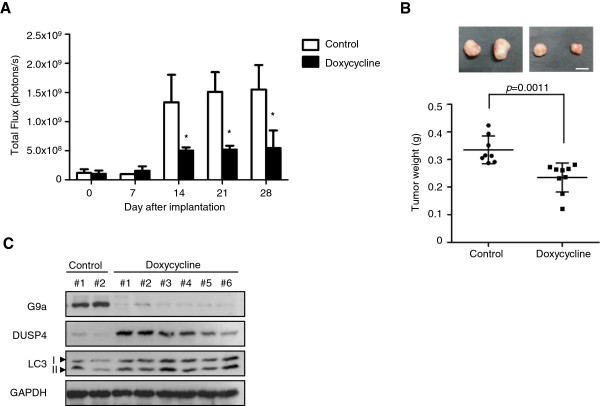
**Inhibition of G9a induces autophagy and displays anti-tumor effects in a xenograft mouse model. (A)** FaDu cells stably expressing luciferase-containing vectors with inducible-knockdown of G9a were orthotopically injected into mice. Tumors from eight mice fed with a control diet or nine mice fed with a diet containing doxycycline were quantified by measuring photon influx. A significant difference was determined between the groups (*, *p* < 0.05). **(B)** Tumors of mice were weighed after sacrifice. **(C)** Tumor lysates were subjected to western blot to analyze the indicated proteins.

To assess the relevance of autophagy in a tumor setting, we established a mouse xenograft model by orthotopic injection of FaDu cells stably expressing Dox-inducible shG9a transgene. Two weeks after implantation, 17 mice were randomly grouped into two sets, fed them with either a normal diet or a doxycycline-containing diet. The results revealed that bioluminescence intensity (Figure 5A) and tumor weight (Figure 5B) both decreased in the mice fed the diet containing doxycycline. Furthermore, induction of G9a-knockdown increased LC3-II and DUSP4, but decreased p62 expression (Figure 5C). Taken together, these findings reveal the importance of G9a in regulating the growth of HNSCC. Knockdown of G9a or inhibition of its catalytic activity induced DUSP4 transcriptional activation, which would inactivate ERK signaling to cause autophagic cell death.

## Discussion

Alteration of epigenetic patterns, which cause oncogene activation or silencing of tumor suppressor genes, has been considered a vital molecular event in the progression of HNSCC [28]. The polycomb group protein enhancer zeste (EZH2), a specific methyltransferase for H3K27, has also been demonstrated to be up-regulated in HNSCC and is related to the growth and ability to metastasize via induction of epithelial-mesenchymal transition gene transcription [29,30]. Furthermore, histone deacetylase enzymes (HDACs) deacetylating amino acid residues in the histone tail are abundantly expressed in HNSCC and function as a modulator for cancer cell growth through negatively regulated insulin-like growth factor binding protein (IGFBP) [31]. Indeed, the HDAC inhibitor, romidepsin, is now being applied in phase II clinical trials of patients with HNSCC. Although its clinical efficacy is still less than optimal, these studies nonetheless provide valuable epigenetic targets for future anti-cancer strategies [32]. In this study, we identified the clinical significance and the predominant role of histone methyltransferase G9a depletion or inactivation of G9a, which suppresses in vitro cell growth and in vivo tumorigenecity, findings that suggest G9a provides the growth advantage of HNSCC. These findings not only support the possibility that the reversibility of the epigenetic process may be a plausible way to destroy cancers, they also provide another target of epigenetic regulators for drug treatment of HNSCC.

This study found that G9a expression is higher in tumor tissues compared to normal adjacent tissues and is significantly correlated with Ki-67 proliferation markers. Interestingly, we also noticed that G9a and Ki-67 present similar distribution patterns, centralized at the basal layer, in the proliferative compartment for normal epithelium [33], but not in other squamous cells in the normal epidermis (Figure 1A and Additional file 5: Figure S4). These findings point to the hypothesis that maintenance of high G9a expression levels may be necessary for both normal basal layer cells and tumor cells to sustain their highly proliferative properties. This phenomenon is further supported by the dramatic decrease of cell proliferation after G9a depletion or enzymatic inhibition in HNSCC cells. G9a plays an important role in the organization of chromatin and regulation of gene expression. Similarly, recent reports have also demonstrated that inhibition or deletion of G9a reduced the proliferation rate of normal bronchoepithelial NHBE cells [34] and resulted in defective development [6,35]. These data also suggest that the proliferative effects of G9a are highly dependent on its histone methylatransferase activity. Indeed, G9a was previously found to epigenetically suppress *p21* mRNA transcription in fetal pulmonary arterial smooth muscle cells, which is an important G1 phase CDK inhibitor [36]. Accordingly, our microarray analysis also found that knockdown of G9a up-regulates *TP53* but down-regulates *CDK* transcription in HNSCC cells. Tumor p53 is a sequence specific transcription factor. Its transcription activity is critical for tumor suppression [37,38]. Recently, it has also been found that G9a inactivates p53 in a transcriptionally independent manner. Other study provides evidence that the G9a-GLP complex methylates p53 protein on Lys^373^ residue and inactivates the growth suppression function of p53 in breast and lung cancer cells [8]. Although the underlying mechanisms of HNSCC remain to be explored, it is possible that G9a epigenetically directs the growth advantage for cancer cells by suppressing key regulators in apoptosis or the cell cycle through multiple routes.

Autophagy has emerged as an important stress response induced by nutrient depletion or drug treatment. Key molecules that modulate autophagy, such as AMPK and TSC1/2, are known to be important in regulating cell survival and proliferation in different kinds of cancer cells [16]. Recently, G9a was also identified as involved in the regulation of the autophagy response. However, there seem to be multiple mechanisms underlying G9a-regulated autophagy and may involve tissue specific pathways. A previous study has shown that pharmacological inhibition of G9a induces autophagy under hypoxia in pancreatic cancer cells via transcriptional upregulation of *BNIP3*, a pro-apoptotic member of the Bcl-2 family [39]. In osteosarcoma cells, autophagy is induced by G9a suppression, which then causes down-regulation of serine-glycine biosynthetic genes *PHGDH* and *SHMT*, suggesting that a G9a-dependent epigenetic program in the control of cell metabolism sustains cancer growth and survival [40]. Furthermore, prior studies also provide evidence that inhibition of G9a leads to reactive oxygen species (ROS) accumulation and then activates autophagic cell death in breast and colon cancer cells [15]. Oxidative stress has been demonstrated to promote autophagy through multiple pathways, including induction of ER stress, NF-κB activation and mTOR inhibition [41]. Similarly, we also noted that knockdown of G9a in both HNSCC and colon cancer cells increased endogenous ROS generation, although its role is not elucidated in this study. Therefore, we suggest ROS may also likely be involved in G9a inhibition-induced autophagy in HNSCC. In addition, another study provided evidence that G9a directly binds to the *LC3* promoter in naïve T cells to execute transcriptional silencing of *LC3*. Thus, inhibition of G9a would activate *LC3* expression and initiate autophagy [42]. However, it may not induce p62 decreasing autophagic flux, a finding that differs from our study (Figure 3D and G). G9a has been demonstrated to cooperate with different transcription factors at chromatin regions to trigger epigenetic regulation for maintaining various physical functions [43]. It interacts with C/EBPβ to regulate *PPARγ* expression during adipogenesis [44]. Furthermore, it may also bind with YY1 to suppress *JAK2* to block leukemogenesis [45]. Therefore, we suggest it is possible that the complexity of the G9a-associated transcriptional complex may cause a diversity of autophagy mechanisms in various tissues.

DUSP4, also known as mitogen-activated protein kinase phosphatase-2 (MKP-2), is a dual serine-threonine/tyrosine phosphatase that specifically inactivates JNK, p38, and ERK [46,47]. A previous study showed that DUSP4 is widely expressed in different tissues and implicated in cancer development. Its expression is down-regulated in serous carcinomas, in contrast to ovarian serous borderline tumors and action through uncoupling activation of the RAS-RAF-MEK-ERK-MAPK pathway, which is a typical survival signal pathway in cancers [48]. Decreased expression of DUSP4 is associated with advanced tumor stage, lymphatic and vascular invasion, and liver and lung metastases in colorectal cancer. [49]. In addition, deregulation of DUSP4 activity contributes to sustaining ERK signaling, resulting in oncogenic transformation in lung cancer cells [50]. In this study, we reveal that inhibition of G9a attenuates cell proliferation through a DUSP4-mediated pathway, consistent with the above literature supporting that DUSP4 represents a tumor suppressor. Furthermore, we also provide substantial evidence that instead of activating AMPK, ERK dephosphorylation by DUSP4 is the major mechanism of autophagic cell death in HNSCC cells and is triggered by G9a inhibition (Figure 4E and F). ERK inactivates tuberous sclerosis (TSC) complex, leading to mTOR activation, and then promotes its downstream target S6K phosphorylation on Threonine 389 (T389) residue, one of the common signals controlling cellular growth and autophagy inhibition [27,51]. Consistent with previous findings, we observed that ERK inactivation and S6K T389 dephosphorylation in G9a-inhibited cells in HNSCC (Figure 4A and Additional file 2: Figure S1), implying that G9a may activate ERK-TSC-mTOR signaling during HNSCC cell growth. Once G9a is suppressed, it leads in turn to mTOR inactivation and gives rise to the autophagic flux.

## Conclusions

In summary, our findings demonstrate that G9a plays a vital role in controlling the switch of growth and death signals in HNSCC. Inhibition of G9a elicits autophagic cell death via a DUSP4-dependent ERK inactivation mechanism, suggesting that targeting G9a is a plausible way to induce death initiation signaling in HNSCC. This study not only sheds light on a novel downstream mechanism regulated by G9a but also provides proof for the possibility that targeting G9a may offer an additional avenue for curing HNSCC.

## Materials and methods

### Cell cultures

The FaDu (ATCC HTB-43, human pharyngeal squamous cell cancer) cell line was purchased from American Type Culture Collection (Manassas, VA) and the SAS (human tongue squamous cell cancer) cell line was obtained from Japanese Collection of Research Bioresources (JCRB cell bank, Japan). The two cell lines were maintained in RPMI-1640 and Dulbecco’s modified Eagle medium (Bibco BRL, Grand-Islands, NY, USA) supplemented with 10% fetal bovine serum, 2 mM L-glutamine, 100 U/ml penicillin and 0.1 mg/ml streptomycin at 37°C in a humidified atmosphere with 5% CO_2_.

### Chemicals

G9a enzymatic inhibitors, BIX-01294 and UNC0638, autophagy-inhibitors, 3-methyadenine (3-MA) and chloroquine (CQ) were purchased from Sigma–Aldrich, St. Louis, MO, USA. MAPK/ERK kinase inhibitor, U0126, was purchased from Cell Signaling Technology, Danvers, MA, USA.

### Immunohistochemistry (IHC)

The study was approved by the National Taiwan University Hospital Ethics Committee. Paraffin-embedded tumor blocks were dewaxed and pretreated in citric acid buffer solution (pH 6.0) (for ki67 staining) or boric acid solution (for G9a staining) by microwave boiling for 15 min to retrieve the antigens. Sections were then incubated with 1% H_2_O_2_ in methanol for 15 min at room temperature to block endogenous peroxidase; they were then blocked with 3% bovine serum albumin for 1 h at room temperature. Staining was performed at 4°C overnight with the indicated antibodies, anti-G9a (R&D Systems, Minneapolis, MN, 1:100) and Ki-67 (GeneTex, 1:100). Sections were incubated with immunoglobulin G (IgG)-biotinylated secondary antibodies (BioGenex) for 40 min then incubated with streptavidin peroxidase (BioGenex) for 30 min at room temperature. Visualization was achieved using 0.03% diaminobenzidine tetrahydrochloride substrate for 3 min, and then sections were counterstained with Mayer’s hematoxylin and mounted. We classified staining intensity having as low (negative and focal expression less than 20% of tumor cells) and high (diffuse expression in more than 20% of tumor cells) expression.

### Lentiviral infections

Lentiviral constructs expressing G9a shRNA, DUSP4 shRNA or control luciferase shRNA were purchased from the National RNAi Core Facility Platform in Academia Sinica, Taipei, Taiwan. The lentiviruses were prepared by co-transfecting shRNA-expressing plasmid, pCMVdeltaR8.91 and pMD2.G into 293 T cells using calcium phosphate. The supernatants were harvested and used to infect cells in medium, which contained 8 μg/ml polybrene (Sigma–Aldrich). Cell medium was replaced and fresh growth medium was added after 24 h. Cells were harvested for the following experiments 72 h later.

### Lipofectamine transient transfection

Cells were transiently transfected with pcDNA3.1-G9a-HA or catalytically inactive dominant negative G9a (pcDNA3.1-G9a-DN-HA) using Lipofectamine 2000 reagent (Invitrogen, Carlsbad, CA, USA) in serum- and antibiotic-free medium. Eight hours later, we changed the media to growth medium and incubated them at 37°C in a humidified atmosphere with 5% CO_2_ for 72 h.

### Western blot analysis

Cells were washed with ice-cold phosphate-buffered saline (PBS) and then solubilized with protease inhibitor (Upstate Biotechnology, Temesula, CA, USA)-containing RIPA buffer. Lysates were immunoblotted with indicated antibodies, anti-G9a, anti-DUSP4, anti-p-AMPK, anti-AMPK, anti-S6K and anti-p-S6K (T389) (Cell Signaling Technology), anti-H3K9me1 (Millipore, MA, USA), anti-GLP, anti-H3K9me2, anti-histone 3, anti-LC3B (Abcam), anti-H3K9me3, anti-caspase 3, anti-PARP, anti-p62, anti-β-actin anti-GAPDH, anti-α-tubulin (GeneTex), anti-p-Akt, anti-Akt, anti-p-ERK and anti-ERK (Santa Cruz Biotechnology, Santa Cruz, CA).

### Cell proliferation and viability assay

Cells were seeded in 96-well plates (2x10^3^ cells/well). We determined their growth rate and viability by MTT (3-(4,5-Dimethyl-2-thiazolyl)-2,5-diphenyl-2H-tetrazolium bromide) assay (Sigma–Aldrich). The formazan crystals resulting from mitochondrial enzymatic activity on the MTT substrate were solubilized with dimethyl sulfoxide and the light absorbance was measured at 570 nm with a spectrophotometer.

### Clonogenic assay

Cells were seeded in 6-well plates (5×10^2^ cells/well) in 3 ml of growth medium at 37°C in a 5% CO_2_ atmosphere overnight. BIX-01294 or vehicle (sterile H_2_O) was added at concentrations indicated for 24 h. The drug-containing medium was then removed and fresh growth medium was added to keep cells growing for ten days. Colonies were fixed with 100% methanol at room temperature for 10 min and stained with 0.058% crystal violet. Colonies containing more than 50 cells were counted.

### Anchorage-independent cell growth assay

Cells were suspended in 24-well plate (5×10^2^ cells/well) with growth medium containing 0.35% agarose over a 0.5% agarose base layer at 37°C in a 5% CO_2_ atmosphere. Three weeks later we counted the number and the diameter of foci.

### 5-bromo-2’-deoxyuridine (BrdU) incorporation assay

Cells were seeded in 6-well plates (3×10^5^ cells/well). After BIX-01294 treatment, BrdU labeling solution (Roche, Palo Alto, CA) was added into each well for 1 h at 37°C. After fixation by 2% paraformaldehyde for 15 min at room temperature and PBS washing for 3 times, cells were incubated with BrdU antibody (Abcam, 1:250) for 1 h at 37°C. Cells were then washed with PBS and incubated with alkaline phosophatase (AP) conjugated secondary antibody (Abcam) at room temperature for 16 h. After PBS washing, the cells were treated with colorsubstrate solution (Pierce, IL, USA) at room temperature for 20 min. The stained cells were photographed and 5 randomly chosen visual fields were counted to determine the percentage of BrdU positive cells.

### Annexin V and propidium iodide (PI) double staining and flow cytometry analysis

Cells were washed twice with ice-cold PBS and resuspended in staining buffer at a concentration of 1×10^6^ cells/ml. We performed Annexin V and PI staining using the Annexin V-FITC Apoptosis Detection Kit (BD Biosciences, San Jose, CA, USA) according to the manufacturer’s instructions. Stained cells were immediately analyzed by BD LSRII Flow Cytometer.

### Examining autophagosomes with GFP-LC3 using fluorescence microscopy

Cells were transfected with GFP-LC3 (pcDNA-6.2-EmGFP-LC3) expressing plasmid with the Lipofectamine 2000 reagent (Invitrogen) and stable expressing clones were selected by blasticidin (10 μg/mL). The localization of LC3 was examined by fluorescence microscopy (Zeiss Axiovert 200 M).

### Transmission electron microscopy

Cells were grown on plastic coverslips and fixed in Karnovsky's fixative (2% paraformaldehyde and 5% glutaraldehyde in 0.1 M cacodylate, pH 7.4) followed by osmium tetroxide. Samples were then dehydrated in ethanol, infiltrated and embedded with TAAB Low Viscosity Resin (TLV) mixture at 60°C for 24 h and sectioned to 80 nm in thickness on 300 mesh copper slot grids. Analysis was performed by transmission electron microscopy (JEOL, JEM-1400) by the Department of Pathology, National Taiwan University Hospital.

### Affymetrix microarray

Labeled probes for Affymetrix microarray analysis were prepared according to the manufacturer’s instructions. Briefly, mRNA was transcripted into Biotin-labeled cRNA in vitro, and then fragmented and hybridized to Human U133 2.0 plus array (Affymetrix Inc., Santa Clara, CA). DNA chips were scanned with the Affymetrix GeneChip scanner, and the signals were measured by GeneSpring GX software.

### Quantitative real-time PCR analysis

RNA from cells was extracted using Trizol (Invitrogen) following the acid guanidium thiocyanate-phenochloroform method. The RT-PCR was performed according to the manufacturer’s protocol using the SuperScript III First-Strand Synthesis System (Invitrogen). We performed real-time PCR experiments using SYBR green reaction mix (KAPA, Woburn, MA, USA) following the manufacturer’s instructions. Primer sequences used are shown in Additional file 6: Table S1.

### Xenograft tumorigenesis model

This study used seventeen non-obese, severly diabetic, female mice with immunodeficient (NOD-SCID) (6–8 weeks old) housed under pathogen-free conditions. In the orthotopic implantation model, FaDu stable clones with inducible-knockdown of G9a expressing plasmid (1×10^6^ cells/mouse) are injected into the upper left side of the month and fed with a control diet. Two weeks later, one group of mice was fed with a doxycycline-containing diet to induce knockdown of G9a. Tumor imaging was performed by intraperitoneal administration of 150 μg/ml luciferin (Biosynth, A.G., Switzerland) and bioluminescence technology (NightOWL LB981 imaging system or Xenogen IVIS-100 imaging system). Photons emitted from specific regions were quantified using Living Image® software (Xenogen Corporation). Six weeks after implantation, the mice were sacrificed and the tumor weights were measured. The use of animals for this study was approved by the National Taiwan University College of Medicine Institutional Animal Care and Use Committee.

### Statistical analysis

All in vitro experiments were performed at least three times. The results are presented as the mean ± SD. One-tailed Student’s *t*-test was performed to analyze differences between experimental groups. Statistical analysis of clinical pathological correlations was performed using the chi-square exact test. All statistical tests included two-way analysis of variance. A *p* value of <0.05 was considered statistically significant in all cases.

### Accession numbers

The NCBI Gene Expression Omnibus (GEO) accession number for the microarray data reported in this paper is GSE56330.

## Competing interests

The authors declare that they have no competing interests.

## Authors’ contributions

Conception and design: KTH, MLK and CTT. Experiments and data analysis: KCL, YSL and KJY. Clinical pathology analysis: CYS. Provision of animal study: MH. Writing and reviewing the manuscript: KCL, KTH, MLK and CTT. All authors read and approved the final manuscript.

## Supplementary Material

Additional file 1: Table S2 Correlation between Ki-67 and G9a expression in tumor sections of 108 HNSCC patients. Click here for file

Additional file 2: Figure S1 Inhibition of G9a activity by UNC0638 treatment decreases cell growth and induces autophagy in SAS cells. **(A)** Immunoblot analysis of autophagy marker LC3 expression of SAS cells treated with various doses of UNC0638 (UNC) for 24 h. **(B)** The clonogenic cell survival assay of UNC treated cells (**, *p* < 0.01). Click here for file

Additional file 3: Figure S2 Inhibition of G9a decreases BrdU incorporation in FaDu cells. The DNA synthesis was examined by BrdU incorporation assay. **(A)** The photograph and quantification results of FaDu cells with G9a knockdown for 72 h. **(B)** The photograph and quantification results of FaDu cells with BIX-01294 treatment for 24 h. Scale bar, 100 μm (***, *p* < 0.001). Click here for file

Additional file 4: Figure S3 Inhibition of G9a activity decreases mTOR substrate S6K phosphorylation in FaDu cells. Anti-phospho-p70S6K (T389) immunoblot analysis of FaDu cells treated with various doses of BIX-01294 for 24 h. Click here for file

Additional file 5: Figure S4 G9a co-expressed with Ki-67 in the basal layer of normal tissues sectioned from HNSCC patients. The expression of G9a and Ki-67 proteins were analyzed by IHC staining of normal squamous epithelium from TMA. Click here for file

Additional file 6: Table S1 Primer sequence used for real-time PCR analysis. Click here for file
