# Supplementary material for: Inhibition of G9a induces DUSP4-dependent autophagic cell death in head and neck squamous cell carcinoma
Source: Mol Cancer. 2014 Jul 15;13:172. doi: 10.1186/1476-4598-13-172 (PMC4107555; doi:10.1186/1476-4598-13-172)
Supplement: Additional file 1: Table S2 — Correlation between Ki-67 and G9a expression in tumor sections of 108 HNSCC patients. [file 1476-4598-13-172-S1.pdf]

		G9a expression		
		low	high	Total
Ki-67 expression	low	37	31	68
	high	12	28	40
	Total	49	59	108
				t-test, *p* value <0.0001
