# Supplementary figures and images for: Inhibition of G9a induces DUSP4-dependent autophagic cell death in head and neck squamous cell carcinoma
Source: Mol Cancer. 2014 Jul 15;13:172. doi: 10.1186/1476-4598-13-172 (PMC4107555; doi:10.1186/1476-4598-13-172)

**A**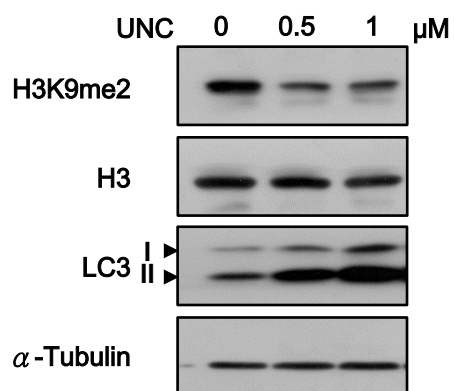**B**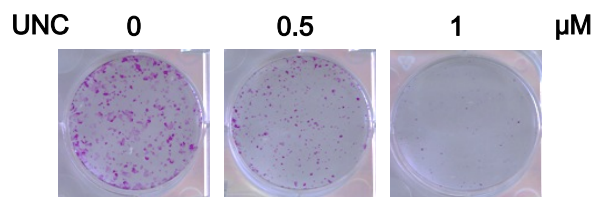

Supplement: Additional file 2: Figure S1 — Inhibition of G9a activity by UNC0638 treatment decreases cell growth and induces autophagy in SAS cells. (A) Immunoblot analysis of autophagy marker LC3 expression of SAS cells treated with various doses of UNC0638 (UNC) for 24 h. (B) The clonogenic cell survival assay of UNC treated cells (**, p < 0.01). [file 1476-4598-13-172-S2.pdf]

**A**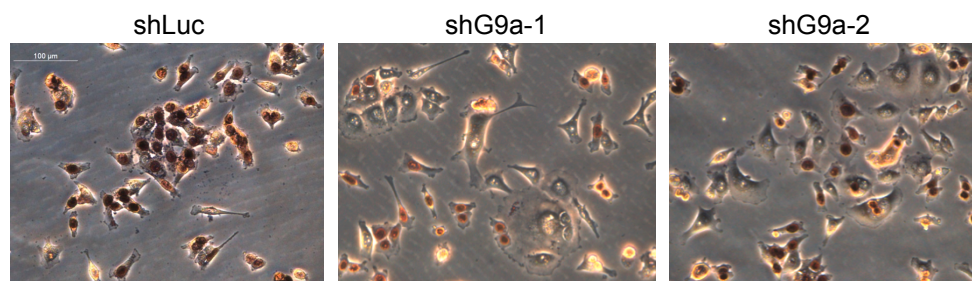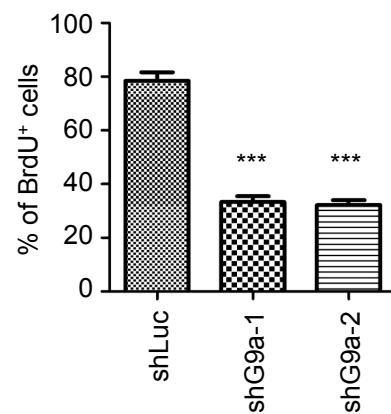**B**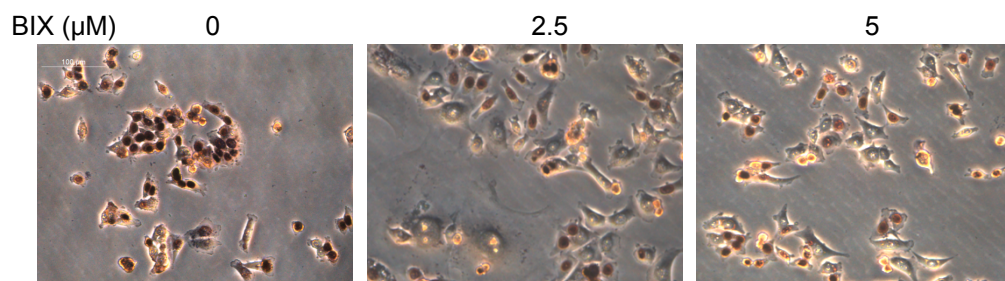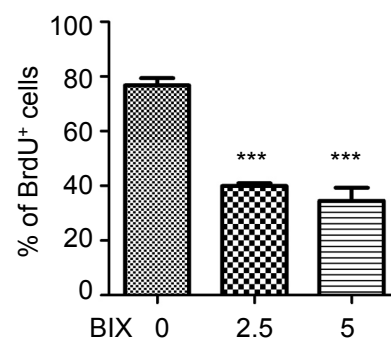

Supplement: Additional file 3: Figure S2 — Inhibition of G9a decreases BrdU incorporation in FaDu cells. The DNA synthesis was examined by BrdU incorporation assay. (A) The photograph and quantification results of FaDu cells with G9a knockdown for 72 h. (B) The photograph and quantification results of FaDu cells with BIX-01294 treatment for 24 h. Scale bar, 100 μm (***, p < 0.001). [file 1476-4598-13-172-S3.pdf]

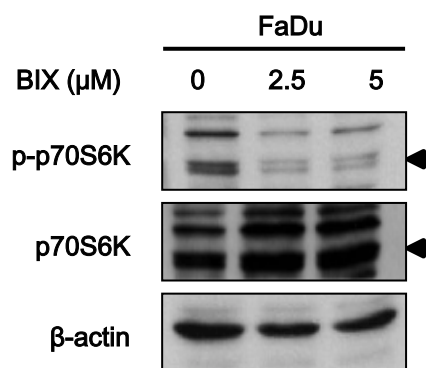

Supplement: Additional file 4: Figure S3 — Inhibition of G9a activity decreases mTOR substrate S6K phosphorylation in FaDu cells. Anti-phospho-p70S6K (T389) immunoblot analysis of FaDu cells treated with various doses of BIX-01294 for 24 h. [file 1476-4598-13-172-S4.pdf]

Ki-67

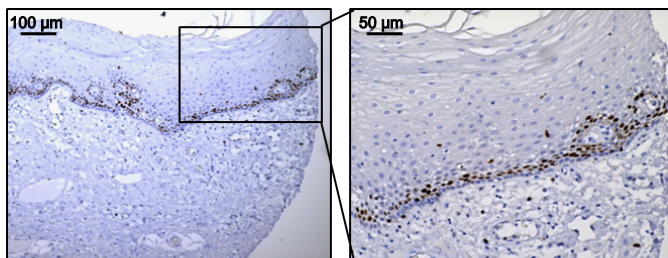

G9a

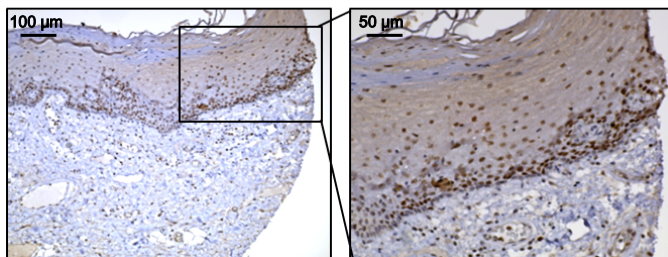

Supplement: Additional file 5: Figure S4 — G9a co-expressed with Ki-67 in the basal layer of normal tissues sectioned from HNSCC patients. The expression of G9a and Ki-67 proteins were analyzed by IHC staining of normal squamous epithelium from TMA. [file 1476-4598-13-172-S5.pdf]
