# Supplementary material for: Inhibition of G9a induces DUSP4-dependent autophagic cell death in head and neck squamous cell carcinoma
Source: Mol Cancer. 2014 Jul 15;13:172. doi: 10.1186/1476-4598-13-172 (PMC4107555; doi:10.1186/1476-4598-13-172)
Supplement: Additional file 6: Table S1 — Primer sequence used for real-time PCR analysis. [file 1476-4598-13-172-S6.pdf]

gene	sense	antisense
G9a	CAAGGAGGAGGACGGTTCCAC	TCGATGTGCTTGTGCTCTGC
DUSP4	GTACAAGTGCATCCCAGTGGA	CTTCATCATCAGGTAGGCCAGG
GAPDH	ATCAGCAATGCCTCCTGCAC	GTGATGGCATGGACTGTGGTC
